# Hand-to-hand combat in the 21st century—INNOAGON warrior or modern gladiator?—a prospective study

**DOI:** 10.3389/fspor.2024.1383665

**Published:** 2024-04-25

**Authors:** Artur Kruszewski, Ilia Cherkashin, Marek Kruszewski, Elena Cherkashina, Xiaoquan Zhang

**Affiliations:** ^1^ Department of Individual Sports, Jozef Pilsudski University of Physical Education in Warsaw, Warsaw, Poland; ^2^ School of Physical Education, Hainan Normal University, Haikou, Hainan, China; ^3^ College of Sports Science, Shenyang Normal University, Shenyang, China

**Keywords:** physical culture, physical activity, sport, hand-to-hand combat, mental health

## Abstract

**Introduction:**

In being an important lifestyle factor, the physical activity influences people's health status, including life expectancy. Specific forms of physical activity are exercises related to hand-to-hand combat in the broadest sense, often overlapping and containing similar elements associated with combat sports (for Western culture) and martial arts (for Far Eastern culture). There are many types of forms of practising hand-to-hand combat exercises, which can be seen as opposing or in some ways complementary, e.g.: “traditional”—e.g., karate or kung fu, practised for hundreds or even thousands of years, usually adhering to moral codes of conduct, and drawing ideas from philosophy, religion vs. “modern”—e.g., American pro wrestling or mixed martial arts—MMA, suggesting a combination of various traditional forms of martial arts. In terms of shaping a healthy lifestyle with mental health elements based on the implementation of hand-to-hand combat exercises, an analysis of these fundamental insights into physical activity is warranted. This study aims to investigate the development direction of the hand-to-hand exercises in Western culture, opposing the “traditional” and “modern” forms.

**Discussion:**

In this study, INNOAGON theory responds to the overuse of the term “science of martial arts”. It is currently being used to promote extreme aggression and violence as an acceptable means of achieving success, and this model is expressively permeating other spheres of social interaction. In INNOAGON's understanding, hand-to-hand combat should be associated with the ancient thought of “doing good” (ancient Greece) or “wisdom” (Far Eastern cultures) including aesthetic and philosophical aspects. This links to numerous psychosocial benefits, self-esteem, discipline, body-mind coordination including breath control and relaxation, respect for others. The modern form of hand-to-hand combat, framed in these discussions as MMA or American pro wrestling, historically refers to the gladiatorial fights held during the Roman Games. The holding of fights in so-called “cages” refers to the arena in which Roman slave fights were held, and the athletes performing there are often referred to as modern-day “gladiators”.

**Conclusion:**

Proper education, including education in the area of physical culture, leading to the development of a society turned towards values broader than just fun or self-satisfaction should be the basis for the development of the next generations. It actually remains to decide which path of social change we will choose: in the case of INNOAGON a positive one, and in the case of modern gladiator a destructive one. Only a proper education combined with a broad programme of social role modelling, social facilitation—the foundations of which are laid by INNOAGON—including promotion on the Internet and social media can provide the right counterbalance to the pathology increasingly penetrating our lives, leading to the promotion of aggression and threats to physical and mental health.

## Introduction

In terms of theory, physical culture should be interpreted as a totality of activities that are in line with the rules and norms of behaviour adopted in a given social environment. The main assumption of this kind of implication is the formation of attitudes oriented towards health-promoting values. Physical culture is defined as the totality of activities in line with rules and norms of behaviors adopted in a given social environment (1). Physical activity as part of a lifestyle is one of the important factors influencing the health status of the population, including life expectancy. This relationship is not unique, but is linked to the environment in which we live, modern health care or genetic factors—beyond our control. Of all these factors, the one that we can most influence is our lifestyle. It is shaped by appropriate patterns of behaviour, both through upbringing and socialisation. To the greatest extent this occurs in the early years of a child's life as a result of interactions within the family and during adolescence as a result of interactions with peers (2).

The benefits of undertaking physical activity are obvious. Physical activity is one of the most important categories for the proper functioning of the human body. Systematic physical activity is a fundamental biological determinant of physical and mental health. Many factors (e.g., environment, healthcare, genetics) mediate this relationship and influence our lifestyle, which is notably shaped by specific behaviour patterns including family and peer interactions during childhood and adolescence, respectively (3).

A proper lifestyle also determines our mental health, which plays an essential role in many individual aspects of life (4, 5).

Operationalized as a set of complex constructs related to self (e.g., self-concept, body image), ill-being (mismanaged emotions, behavioural disorders), well-being (positively framed states and skills) and cognitive functions (with additional brain health mechanisms), the mental health plays an essential role in many individual aspects of life. For instance, whilst a person with a balanced self-esteem and a healthy body image is more resilient to stress and can cope better with life issues, individuals with cognitive challenges are often socially isolated and more prone to negative thought patterns and dysfunctional behaviours (5, 6).

Used in a moment of danger to slow down destructive agents, to survive or to maintain health, hand-to-hand combat is a form of physical activity often overlapping and containing elements associated with combat sports (7) and martial arts (8–12). Hand-to-hand combat is a key phenomenon used in a moment of danger leading to the slowing down of destructive agents, the drive to survive or maintain health (13).

It is therefore an ideal form of exercise that is accessible to everyone regardless of age or fitness level. In addition to the objectives commonly recognised in Western society, namely the combat and self-defence element (14, 15), hand-to-hand combat training integrates movements of the whole body. It combines breathing techniques, correcting postural changes and repetitive balance practice into a coherent whole, complementing preventive philosophy. In Far Eastern countries, elements of hand-to-hand combat have been practised for centuries by people of all ages because of their health benefits (16).

There are many types of forms of practising hand-to-hand combat exercises, which can be seen as opposing or in some ways complementary, such as:

“Hard”—e.g., karate and boxing, focusing on delivering blows or hitting the opponent vs. “soft”—e.g., Aikido, using the opponent's Energy to defeat the opponent

or “Traditional”—e.g., karate or kung fu, practised for hundreds or even thousands of years, usually adhering to moral codes of conduct, and drawing ideas from philosophy, religion or educational teaching vs. “Modern”—e.g., American pro wrestling or mixed martial arts—MMA, often suggesting a combination of different traditional forms of martial arts (17).

In terms of shaping a healthy lifestyle with mental health elements based on the implementation of hand-to-hand combat exercises, an analysis of these fundamental insights into physical activity is warranted. This publication aims to discuss the evolution of hand-to-hand combat practices in Western culture, specifically examining the contrasting perspectives of “traditional” vs. “modern” approaches, epitomized by the dichotomy of “warrior or gladiator”.

### The INNOAGON warrior

INNOAGON—an acronym for innovative agonology, is an applied science focusing on promotion, prevention and therapy addressing all dimensions of health and optimising actions enhancing survival (from micro to macro scale) (18).

The INNOAGON theory acknowledges the pivotal role of physical culture in shaping health-promoting behaviors. It also underscores the integration of physical and mental development within martial arts, which fosters adherence to a distinct behavioral philosophy.

Out of this broad spectrum of the impact of INNOAGON theory, the issue of teaching and applying hand-to-hand combat in modern society is brought to the foreground. By training in hand-to-hand combat, we shape self-defence skills, develop mental toughness, increase self-esteem and self-confidence and generally influence a change in life attitudes (18).

Meanwhile, the promotion of neo-gladiatorism (bloody spectacles) under the camouflaged name of “mixed martial arts” began to dominate the internet and electronic media. Thus, the continued use of the term “science of martial arts” has begun to lose its meaning for two reasons of equivalent importance. Firstly, the seriousness and social mission of science is being devalued. Secondly, scientific journals (and it does not matter if they are evaluated by WoS or other prestigious literature databases) that have the name “martial arts” in their title are at risk of numerous manipulations. Among the visible ones “to the naked eye” is the use of the phenomenon of “mixed martial arts” in the propaganda of extreme aggression and violence as an acceptable way to achieve success, and this model permeates expressively other spheres of social interaction. The threat of instrumental use of the “science of martial arts” in this practice is clear (18).

In the general approach of the INNOAGON theory, the promotion of physical culture taking into account its health-promoting effects has its origin in the ancient hand-to-hand combat systems of both the Far East and the West, for which the preservation of the balance of the universe is a fundamental element leading to proper development (13).

In Far Eastern culture, the source of the complex effect on body and mind is the Yin Yang symbol. As a recognised symbol of Taoism, its symbol owns a philosophical rather than a religious significance. It derives from the text of the Tao Te Ching, which translates as “the way of honesty”. The title can mean “The Book of the Way and its Virtue”. In its eighty verses, it contains a treatise on how to live in the world with goodness and honesty. The text is attributed to Lao Tzu (or Laozi, which literally means “old master”), a chronicler of the Zhou dynasty in the sixth century BC, and is a fundamental text of philosophical Taoism (19).

Yin is the feminine element and implies the qualities of slow, soft, non-substantial, diffuse, cold, wet and calm, and is associated with water, earth, birth and birthing. Yang is the masculine principle and implies the qualities of fast, hard, solid, dry, focused, hot and aggressive, and is associated with things like fire and sun. When someone is calm, relaxed and accepting of everything around them, they are in a yin state. When someone is tense like a coiled spring and completely focused on one thing, they are in a yang state. Neither of these is good or bad and both are appropriate in their place. These distinctions do not imply any observed or prescribed gender attitude behaviour, but are philosophical or linguistic distinctions (19).

These opposites revolving around each other create momentum and the harmony resulting from balancing them is essential. They have their practical applications, of course; the transition through yin and yang in movements is evident in offensive and defensive balance, but also in the development of a calm mind that can think clearly even during violent actions. This learned transition is a skill that eventually becomes so intuitive that the principle permeates all areas of life. The balance of yin and yang forms the basis of traditional Chinese medicine such as Tai Chi and numerous martial schools (18).

In a historical sense, hand-to-hand combat—in European culture, is associated with the ancient form of wrestling as the first sport accumulating the most important of the recommended health benefits of physical activity. Offering, in a utilitarian sense, gentle and relatively mild ways of restraining an opponent's movements, and thus also fulfilling the criteria of “equitable self-defence” (20).

In recent decades, an increasing number of studies have confirmed the therapeutic effects of hand-to-hand combat training on various aspects of health, such as improved balance control (21–23) or improved mental health in older people (24). The therapeutic value of using elements of hand-to-hand combat has also been noted in reducing stereotypic behaviour in children on the autism spectrum (25, 26).

Combat training activities can offer adaptable exercise therapy suitable for individuals of diverse fitness levels and ages, promoting holistic health benefits. Tailoring exercise therapy specifically for older adults and children with clinical conditions is essential, as these groups often face greater obstacles in participating in physical activity compared to healthy young and older individuals (27).

The fundamental element differentiating the group of combat sports, within the Olympic family, is their links with the philosophy and pragmatics of hand-to-hand combat. A characteristic feature of all these disciplines is the complementary impact on the organism of a young adept of hand-to-hand combat (e.g., on the somatic, motor, intellectual, moral, emotional spheres). It should be emphasised here that this possibility is rooted, on the one hand, in the ancient slogan of the harmonious shaping of spirit and body and, on the other, in the necessary competence of the teacher. If these two criteria are fulfilled, among advanced athletes, mental development often becomes more important than the achievement of sporting success alone (28).

Although hand-to-hand combat can be associated with aggression or violence, in INNOAGON terms, it combines ethical and philosophical aspects that can overlap with some holistic movement-based mind-body disciplines such as yoga and qigong. They are linked to a number of psychosocial benefits, including discipline, respect, self-esteem, self-respect breath control and relaxation, body-mind coordination (29–31). In the numerous scientific studies developed, hand-to-hand combat is described as a useful instrument for the effective management of physical and mental energy, but its psychotherapeutic applications are also indicated (30, 32–35). Among its many impacts, it is indicated to have a positive effect on mental health outcomes and to improve well-being and reduce symptoms associated with internalising mental health problems, such as anxiety and depression (36). An extremely important part of understanding the aspects that may affect mental health outcomes is a broad approach that includes the structure of martial practice, its tasks, types, settings, guidelines, etc. (32, 10). Therefore, in order to better understand the broader relationship between physical activity based on broadly defined activities involving elements of hand-to-hand combat and mental health and body and spirit formation, moderating factors and mediating mechanisms need to be considered (30, 37). The knowledge gained in this way, which will take into account the complexity of issues arising from properly conducted hand-to-hand combat training, can inform mental health interventions, strategies and policies. However, it is important to remember that the relationship between training with hand-to-hand combat elements and mental health is complex and multifaceted, covering a very broad spectrum of impacts. Many elements still need to be elaborated and understood, including in such a seemingly obvious matter as definitions (38) or cognitive factors including socio-cultural and environmental aspects (39, 40).

### The modern gladiator

Contemporary forms of hand-to-hand combat sports are constantly changing and modifying, with new, more or less popular ways of competing emerging. Undoubtedly, examples of such forms include MMA (Mixed Martial Arts), Pride, K1, etc. A notable departure from the values underpinning the values of modern Olympism is the encroaching glorification of aggressive behaviour as proper to forms of pseudo-sports competition (21).

These modern forms of hand-to-hand combat sports find the main justification for their activity in the financial result. According to statistics, the highest income in the area of martial arts business was recorded in the USA, Canada, Australia, the UK and the European Union. It is significant that there are no countries from the Far East in this ranking (41).

Currently, approximately 3.6 million people in the US actively participate in martial arts industry activities each year. It should be pointed out that people under the age of 6 have not been included. Of this group, 35% of those interested in martial arts attend MMA classes. This is followed by karate (22.2%), taekwondo (12.8%) and judo (10.2%). Taking the bottom line into account, the highest earning discipline in the US is MMA, bringing in an average of US$254,083 in annual revenue, with boxing coming in second and Brazilian jiu-jitsu third. Disciplines that are associated with Far Eastern martial arts take further places in this statistic, karate (ranked fourth), taekwondo (fifth) and kung fu (sixth) (41).

According to a study by Trebicky et al. (42), the presentation of aggressive behaviour is becoming an important element of fighting tactics among MMA fighters. A study on the analysis of external appearance indicative of aggressive behaviour indicated a significant association with the percentage of fights won.

An essential element in presenting an aggressive appearance was the significant effect of an apparent “aggressive gaze”, which is achieved by a horizontally narrowed eye-surround, presumably due to developed male features such as prominent eyebrow arches. Converging results are presented by other authors, who found that physiological facial arrangement is a reliable indication of a tendency towards aggression. Other facial features, including the shape of the nose, mouth, chin and especially the eye area, contribute significantly to perceived aggressiveness and potentially reflect the likelihood of winning. In addition, combat history experiences, such as scars and wounds, can also be strongly linked to aggression (43–45).

The appropriate level of musculature and masculinity as well as the aggressive behaviour so desired by MMA fighters correlates with testosterone levels. Research by Lassek and Gaulin (46) indicates that the specific facial configuration in heavyweight athletes may be due to the influence of testosterone and other hormones affecting growth. Also, this thesis is supported by the results of a study of sumo wrestlers, where an index of prenatal testosterone intake resulting in a negative relationship between the ratio of the second and fourth toes correlates with the performance of Japanese athletes (47).

Similar requirements indicating the importance of outward appearance are also noticeable in the professional form of American wrestling (pro wrestling). This is a form of competition combining real combat in a face-to-face clash with a media performance in an arena, presenting a predetermined scenario. Satisfying media needs and the desire to make the performances more appealing means that high-risk situations are increasingly being introduced. Individual scenes and even scenarios have been linked to the personalities of individual wrestlers, making violence central to both competitive and show outcomes (39, 40). This has the effect of putting the athletes involved in these shows at risk of losing their health, even in the form of concussion.

The importance of mental preparation, a characteristic of elite athletes, such as mental toughness, self-confidence (48), motivation (49), goal-setting, and mood states (50), is reflected and becoming increasingly important in managed careers among MMA fighters and American pro wrestling athletes. There is also emerging research indicating an increase in the importance of guided competition strategies related to aggression leading to success (51, 10).

We are currently seeing an increase in the importance of sport psychology in the appropriate development of sporting careers. Given the violent nature of MMA, the repeated references to the visibility of violent behaviour and fear, issues of the impact of this type of projection on young society are becoming an important element of social projection. It is suggested that MMA participants should take the time to learn self-regulation skills, explore their fears and understand the potential financial stresses associated with an MMA career. Similarly, coaches of MMA fighters should understand that they should emphasise mental skills in the talent development process (52).

## Discussion

Historically, the common perception of the elements of hand-to-hand combat has been linked to a broad impact in both the physical and mental spheres. In Far Eastern culture, the elements of hand-to-hand combat guided the development of successive generations, combining physical development with the embodiment of wisdom. This practice, through the unique pupil-master bond, is linked to a duty of respect for the elderly, still evident today. In this culture, the elderly are still associated with wisdom. The revival of the modern Games in the 19th century and the creation of the Olympic Charter in 1914 brought a renewal of the Olympic idea, based on the thoughts of the ancient Greek philosophers. This gave a new impetus to the development of the society of Western culture; respect for the opponent, the values of equal opportunities and fair play were to contribute to the mutual respect of all mankind. In the early days, this development was in line with the idea of rediscovered values of the development of spirit and body, kindness and wisdom. Now, once again (as in the era of the Roman Games), widespread social welfare, the pursuit of profit by satisfying the shallow needs of society in the age of the internet and television spectacle have led to the spread of fiction and neo-gladiatorism (21).

It is therefore necessary to indicate a clear separation between the elements of hand-to-hand combat included in the broader promotional process. It can be done through the INNOAGON, by indicating the therapeutic effect of hand-to-hand combat training and touching on various aspects of health from the superficial association of hand-to-hand combat under the name Mixed Martial Arts MMA or American pro wrestling.

Nixon (53) identifies athletes as participants in “sports networks”. By being deeply rooted in these structures, contacts between athletes tend to reinforce each other. This means that different members of this group are likely to affirm similar normative ideas. This mutual reinforcement is explained by a shared “style of thinking.” In this way, the ideas created cause members of these thinking collectives to be reluctant to adopt a different style of thinking.

The hand-to-hand combat associated with many forms of culture must see extremely wide-ranging possibilities for influencing both physical and mental development. Patterns drawn from history are an important source of our learning, so it seems reasonable to argue that now once again (as in the era of the Roman Games), widespread social prosperity, the satisfaction of society's shallow needs is leading to the abandonment of higher values ascribed to man as a rational being. In the age of the internet and television spectacle and the focus only on financial gain, we are becoming a society of leisure, consumption, entertainment and lack of morality.

Therefore, it becomes extremely important to get an answer to the following question—which direction of shaping the style of thinking, from the point of view of social impact, should be promoted in such a wide group of participants and fans of the hand-to-hand combat sports market.

## Conclusion

Proper education, including education in the area of physical culture, leading to the development of a society turned towards values broader than just fun or self-satisfaction should be the basis for the development of the next generations. It actually remains to decide which path of social change we will choose: in the case of INNOAGON a positive one, and in the case of modern gladiator a destructive one. Only a proper education combined with a broad programme of social role modelling, social facilitation—the foundations of which are laid by INNOAGON—including promotion on the Internet and social media can provide the right counterbalance to the pathology increasingly penetrating our lives, leading to the promotion of aggression and threats to physical and mental health.

## Data Availability

The original contributions presented in the study are included in the article/Supplementary Material, further inquiries can be directed to the corresponding author.
